# Low Expression of Occludin in the Melanoma Patient

**DOI:** 10.30699/ijp.2019.85213.1801

**Published:** 2019-09-22

**Authors:** Pouri Salehi, Farzaneh Tafvizi, Kambiz Kamyab Hesari

**Affiliations:** 1 Department of Biology, Parand Branch, Islamic Azad University, Parand, Iran; 2 Department of Pathology, Tehran University of Medical Sciences, Tehran, Iran

**Keywords:** Occludin, Melanoma, Down-regulation, Real-time

## Abstract

**Background & Objective:**

Malignant melanoma is the fatal cutaneous neoplasm which is curable by the early diagnosis. The expression of occludin protein which is an integral membrane protein is altered in an epithelial-to-mesenchymal transition. Although, recent studies provide sufficient evidence supporting the functional importance of occludin in cancer, the prognostic significance of occludin expression levels in melanoma remains obscure. The aim of this study was to determine occludin expression level and its correlation with clinicopathological features of the patients with melanoma.

**Methods:**

The occludin mRNA level was compared between paraffin-embedded tissues of 40 patients with melanoma and 10 subjects with normal skin. The quality and quantity of the RNA was determined and occludin expression level was measured using Real-time PCR and ∆∆CT computational technique.

**Results:**

The occludin mRNA level reduced five-fold in the melanoma patients compared to the control group (*P*=0.000). No significant difference was observed between male and female cases (*P*=0.533). No significant correlation was observed between occludin mRNA level, mitotic count (*P*=0.252), and Breslow levels (*P*=0.171)

**Conclusion:**

We can conclude that down-regulation of occludin expression in the patients with melanoma is a hallmark of cancer progression and it might be used as a prognostic factor. No significant correlation was found between occludin gene expression and clinicopathological characteristics including Clark level, Breslow staging, mitotic count, age and gender (P<0.05).

## Introduction

Cancer is a disease triggered through the genetic mutations which confer the cells unlimited proliferation capacity, loss of response to the growth inhibition factors, apoptosis and angiogenesis (1). Melanoma is the most aggressive type of skin cancer with a lifetime risk about 2% (2). Also, it is an aggressive cancer with an increasing incidence (3). Clinical, histopathological, epidemiological and molecular data indicate that melanoma is comprised of biologically distinct subtypes (4) including spreading melanoma, lentigo maligna melanoma, nodular melanoma and acral lentiginous melanoma (5). The risk factors that contribute to the melanoma are age, gender, chronic exposure to the chemical and physical mutagens, ultraviolet radiation, and genetic factors (6). Melanoma is usually curable by surgery in its early stages, but even though there are recent advances in the development of molecularly targeted therapies against disseminated disease (7). Genetic factors are the most important predictive factors for the melanoma risk and they account for a part of melanoma cases (8).

Tight junctions (TJ), adherens junctions (AJ), and desmosomes are responsible for the contacts between adjacent cells. TJ proteins are the most apical intercellular junctions in the epithelial cells. These proteins are involved in maintaining cell polarity, establishing organ-specific apical domains and also recruiting signaling proteins involved in the regulation of various important cellular functions including differentiation, migration, and proliferation (9). 

TJ comprise transmembrane proteins and cytoplasmic proteins including occludin, claudins and junctional adhesion molecules (10). Occludin and claudins have tissue-specific expression and are crucial for the tight junction barrier function (11).

Occludin knockdown in the intestinal cell lines has been shown to increase macromolecule permeability (12, 13).

It is reported that occludin and claudins are involved in proliferation and differentiation of keratinocytes (14). Occludin is localized at the edge of migrating cells and regulates directional cell migration. Occludin has biological roles with numerous signal transduction molecules (15). This protein is essential for the tight junction formation where occluding-deficient mice had defect in the skin barrier (16). 

Controversial reports exist on occludin expression in different organs. Claudins 1, 2 and 7 are down-regulated while claudin 4 is up-regulated in breast cancer (17). 

Thus, many studies have reported that loss of TJ proteins (including Claudin-7 and occludin) can enhance tumour progression (18, 19).

Down-regulation of occludin is a common feature of epithelial-mesenchymal-transition in the tumors derived from epithelial cells (20). 

Low expression of occludin has been reported for the esophageal squamous cell carcinoma (ESCC) as compared to the adjacent non-neoplastic specimens (21). 

Based on the literature, the scarce information exists on the tight junction proteins in cancers including lung cancer (11). Although recent studies provide sufficient evidence supporting the functional importance of occludin in cancer, the prognostic significance of occludin expression levels in melanoma remains obscure. Also, there is no information on the role of occludin expression in melanoma. Thus, the aim of this study was to determine occludin expression level and its correlation with clinicopathological features of the patients with melanoma. 

## Materials and Methods

**Patients AND Samples**

All Ethical considerations were considered in this study. Paraffin-embedded blocks from 40 patients with melanoma and 10 normal samples (as a control group) were studied after obtaining their written consent. The samples were referred to the Pathology Laboratory of Razi Hospital (Tehran, Iran) during 2013-2016 and were selected after a confirmed diagnosis by a pathologist. All the patients aged between 21-87 years old with no previous use of anti-pregnancy medications, smoking and immune system disorders at least for past 5 years. Demographic and clinicopathological features of the patients including Mitotic count, Clark level, Breslow staging, age, and gender are provided in Table 1. Mitotic count is important part of the staging of melanoma tumor which is evaluated by the pathologist (22).

**RNA E** **xtraction** **and CDNA Synthesis **

The tissue samples were deparaffinized by xylene (1000 µl) at 37ºC for 5 minutes. Then microtubes were centrifuged at 3800 rpm for 5 minute, the supernatants were removed and then 1000 μL of ethanol was added and inverted for 5 minutes. Finally, samples were centrifuged at 13000 rpm at 6ºC for 5 minute and the ethanol and xylene was entirely removed from the microtubes. Total RNA was extracted using the RNX plus™ kit (Cinnagen, Tehran, Iran) based on the manufacturer’s recommendations. A 100 μL of the tissue sample was homogenized with the 500 μL of the RNX-PLUS solution and incubated at room temperature for 5 minutes. Chloroform (200 μL) was added to the solution and centrifuged at 12000 rpm for 15 minutes. The supernatant was transferred to another tube and equal volume of isopropanol was added. The mixture was centrifuged at 12000 rpm for 15 minutes and the resulting pellet was washed in ethanol (70%) and dissolved in DEPC-treated water. The purity and integrity of the extracted RNA was evaluated by optical density measurements and visual observation of sample electrophoresis on 2% agarose gel using NanoDrop spectrophotometer (23). The cDNA was synthesized from total RNA using the commercial kit (c, USA). Each microtube was added with 1 μL of random hexamer (5 µM), 1 μL of oligo (dT) primer (5 µM), 1 μL of deoxynucleotide (dNTP) (10 mM), 5 μL of RNA, 0.5 μL of Moloney murine leukemia virus (MMLV) reverse transcriptase, 2 μL of MMLV buffer, and 9.5 μL of DEPC-treated water. The total volume of the final mixture was expected to reach 20 µL. The samples were incubated at 65°C for 5 minutes and then placed on ice immediately. Afterward, they were run at 42°C for 60 minutes.

**Real-Time PCR**

Real-time (RT)-PCR was performed using the Applied Biosystems 7500 Sequence Detection (USA). In the present study, glyceraldehyde phosphate dehydrogenase (GAPDH) was considered as the housekeeping gene because of its permanent expression in the most cells and tissues (24).

The sequence of specific primers for occludin and GAPDH was retrieved from the National Center for Biotechnology Information (NCBI) website. The specific primers of these two genes were designed using the Primer Express Software and their specificity was blasted in the NCBI. Table 2 presents the sequence of the primers used in this study. Real-time PCR was used to measure the expression of occludin and GAPDH (as the control at mRNA level). A StepOne real-time PCR system was used for the relative quantification through the measurement of fluorescence increase following the application of SYBR Green. The real-time PCR reaction was optimized at the final volume of 20 µl. The reactants included 10 μL of SYBR TM (2X) Master Mix (Takara Company), 10 µM of the reverse and forward primers (Takapoo Zist Co.), 7 μL of deionized water, and 2 μL of the cDNA template. The temperature program of the device was optimized as follows: pre-denaturation at 95°C for 10 seconds; 30 cycles of denaturation at 95°C for 5 seconds; annealing and extension at 60°C for 34 seconds. Each experiment was repeated at least 3 times in order to ensure reproducibility.

The melting curve was drawn through measuring the changes in the fluorescence level at different times using the real-time PCR device. After the amplification reaction using the relative quantitative real-time PCR, the raw data in the form of Ct values were drawn out of the device, calculated through∆∆ ct, and converted into the relative quantity. All the experiments were performed at least in triplicate (24).

**STATISTICAL ANALYSIS**

*T*-test method was used to compare the occludin expression between melanoma patients and control group. The correlation was applied to analyze the association between occludin expression and patients’ clinic-pathological data. Statistical analysis was performed using GraphPad Prism (ver. 6.0) and SPSS 19.0 (SPSS Inc., Chicago, IL, USA) at a significance level of P-value<0.05.

## Results

The demographic and pathological information of the patients is presented in Table 1. According to the results, the mean age of the patients and the control group was 62.84±13.06 and 64.7±9.93 years, respectively. No significant difference was observed on the mean age between normal and melanoma patients (*P*=0.92). 

Also, 11 male and 29 female were included into the study. Based on the Breslow staging, thin melanoma<1 mm was observed in the 62.5% of the patients while 1 mm and non-thin melanoma>1 mm were 5 and 32.5%. Base on the Clark's anatomic level, the I, I-II, II, II-III, III, III-IV, IV, IV-V and VI were observed in 12.8%, 5.1%, 15.4%, 5.1%, 12.8%, 7.7%, 25.6%, 7.7% and 5.1% of the patients, respectively. The site of invasion was in 10.0, 37.5, 17.5 and 35.0% for head, face, upper limb and lower limb, respectively. Mitotic count as high and low were 10 and 90%, respectively. 

**Fig. 1:**
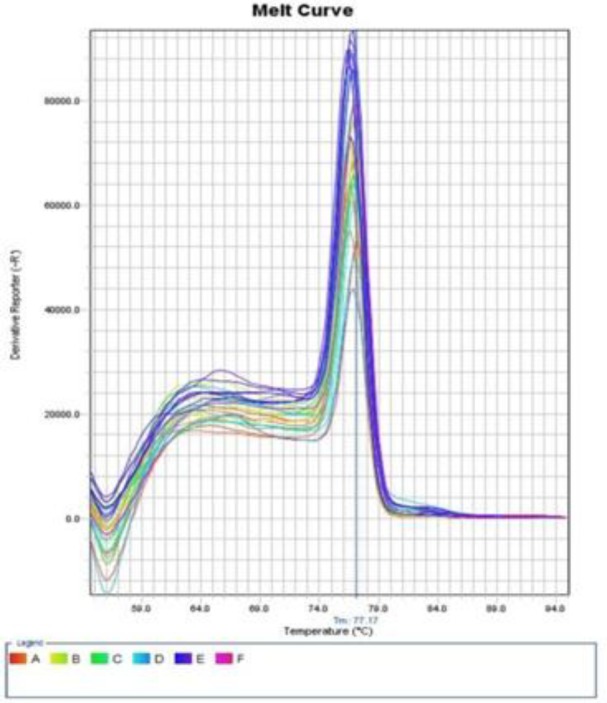
The melt curve of the occludin gene

**Fig. 2:**
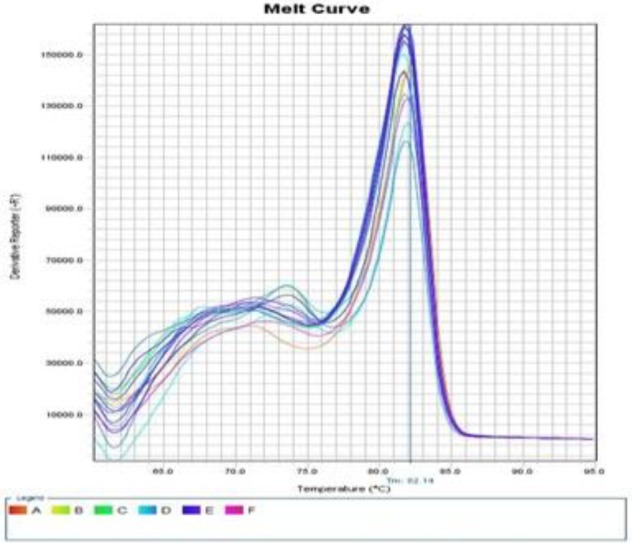
The melt curve of the GAPDH gene

**Fig. 3:**
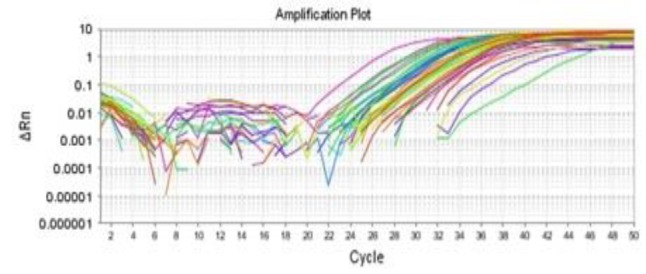
The occludin amplification plot in patients and normal samples

The melt curves of the occludin and GAPDH gene using RT-PCR are presented in Figures 1 and 2. Also, the occludin gene amplification plot in the normal and patient groups is shown in Figure 3.

**Fig. 4:**
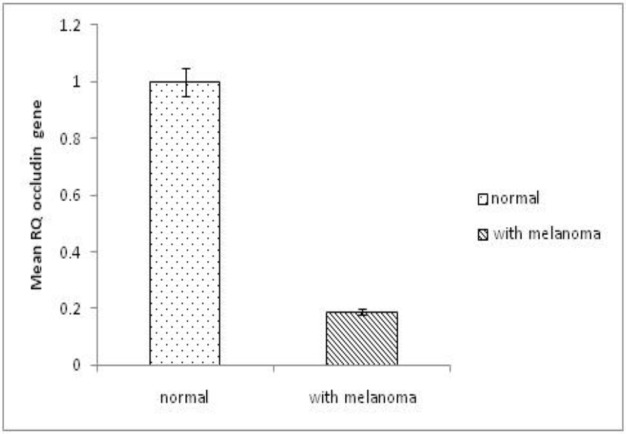
The occludin expression levels in patients and normal samples

Occludin mRNA expression level was 0.19±0.256 lower in melanoma patients than in the normal samples. According to the Figure 4, significant difference was detected on occludin mRNA expression level between the patients and the normal subjects (*P*=0.000). The occludin expression level reduced five-fold in the melanoma patients compared to the control group (*P*=0.000). 

Occludin expression level in males and females was 0.251±0.292 and 0.188±0.242, respectively. No significant difference was observed between male and female in this regard (*P*=0.533). No significant difference was detected on occludin expression in the patients < 60 and > 60 years old either (*P*=0.89).

Additionally, no significant difference was detected on occludin expression based on the mitotic count (*P*=0.252) (Table 3). Based on the Breslow staging, no significant difference was observed between 2 groups (*P*=0.171) (Table 4). No significant association and correlation was observed between occludin expression and clinicopathological characteristics including age (r^2^=-0.047,* P*=0.8), gender (r^2^=-0.15, *P*=0.36), mitotic count (r^2^=0.03, *P*=0.8), Clark level (r^2^=0.013, *P*=0.8), and Breslow staging (r^2^=0.084, *P*=0.613).

**Table 1:** The demographic and clinicopathological features of the patients

		N	(%)
Age	62.84±13.06	40	100
Sex	male	11	27.5
	female	29	72.5
	thin melanoma<1 mm	25	62.5
Breslow Staging	1mm	2	5
	non thin melanoma>1mm	13	32.5
	I	5	12.8%
	I-II	2	5.1%
	II	6	15.4%
	II-III	2	5.1%
Clark's Anatomic Level	III	5	12.8%
	III-IV	3	7.7%
	IV	10	25.6%
	IV-V	3	7.7%
	VI	2	5.1%
	Head	4	10.0%
	Face	15	37.5%
Mitotic Count	High	4	10.0%
	low	36	90.0%
Site of Invasion	Upper limb	7	17.5%
	Lower limb	14	35.0%

**Table 2:** The primers specifications for Real-time PCR

Name		Tm	Amplicon Size
GAPDH F	CCCACACACATGCACTTACC	60	85 bp
GAPDH R	TGCCTGTCCTTCCTAGCTCT	60	
OCLN F	TCCAATGGCAAAGTGAATGA	58.96	84 bp
OCLN R	TCACCAGGGCTGCTTTTAAC	59.53	

**Table 3:** The occludin expression based on the Breslow level

	Breslow				P-value
	non thin melanoma≥1mm		thin melanoma<1 mm		
RQ	Number	Mean ± SD	Number	Mean ± SD	
	15	0.27 ±0.27	25	0.16 ±0.24	0.171

**Table 4:** The occludin expression based on the Mitotic count

**P-value**	Mitotic count				
	High mitotic activity		Low mitotic activity		
	Number	Mean ± SD	Number	Mean ± SD	RQ
**0.252**	4	0.35±0.3	36	0.19±0.25	

## Discussion

Melanoma incidence rate has been increased during the last decades in many countries with white populations. The frequency of melanoma incidence is closely related to the skin color and depends on the geographic zone. Based on the reports melanoma is diagnosed at earlier ages. The mean reported age for melanoma is 55 years. Also, melanoma incidence increases in the individuals older than 65 years (25). In regards to the sex, controversial reports exist where higher melanoma was observed mostly in men in Australia and the United States; however, in countries with a lower incidence, such as Great Britain and Germany, a higher ratio was reported in women (25). The epidemiological evidence implicates that sun exposure and ultraviolet radiation cause damages to the skin DNA which is the key pathogenesis factor for the melanoma tumors (25). Although, direct mechanism of occludin function on the tight junction formation is not fully elicited, it is reported that loss of occludin attenuates the activation of PI3K which can cause actin cytoskeleton disorganization and reduced cell protrusions (15). Therefore, occludin is essential for the leading-edge localization of polarity proteins aPKC-Par3 and PATJ and promotes cell protrusion by regulating membrane-localized activation of PI3K (15). 

It is well documented that melanoma risk is determined through the interplay between the genetic factors and the sunlight exposure (26). 

Based on our knowledge, this is the first report on occludin gene expression in the patients with melanoma. 

In the present study, the occludin mRNA expression level reduced five-fold in the melanoma patients compared to the normal group. This decrement level was significant (*P*˂0.05). No significant difference was observed between male and female on occludin expression and no significant difference was detected on the age (<60 and ≥60 years old) between normal and melanoma patients. No significant correlation was found between occludin expression levels and clinicopathological characteristics including mitotic count, Clark level, Breslow staging, age and gender (*P*>0.05).

In our present study, down-regulation of occludin mRNA was markedly observed in the melanoma as compared to the normal tissues.

Occludin can recruit signal transduction molecules to the tight junctions (10). Down-regulation of the occludin has been reported in gastric cancer, hepatocellular carcinoma and breast cancer (18). Also, down-regulation of occludin has been shown in the most samples of lingual and bronchial squamous cell carcinoma (11, 27). For instance, Rachow *et al*. (2013) reported that down-regulation of occludin decreased cell-cell adhesion and altered epidermal differentiation (28). Down-regulation of some tight junction proteins decreased epithelial proliferation in vertebrate and invertebrate model systems (29). Despite numerous reports on the role of the gene expression in the tight junction, it remains unclear how the modified expression of any given specific tight junction protein is associated with the cancer pathogenesis (11). Animal studies revealed that knock-down of occludin in the breast cancer cells leads to the increased invasiveness (18).

TJ proteins occludin and claudins, junctional adhesion molecules and tight junction plaque proteins ZO-1 and ZO-3 which maintain apicobasal polarity, form dynamic barriers and promote epithelial integrity (30). 

Similarly to the adherens junctions, the interaction between occludin and zona occludens-1 (ZO-1) plays a crucial role in maintaining the structure of the tight junctions, where occludin is linked to the actin cytoskeleton through ZO-1. TJ proteins, e.g. ZO-1, are also observed outside the distinct membrane structures in the cytoplasm, suggesting tight junction-structure-dependent and structure-independent functions. Both ZO-1 and b-catenin appear to be shuttle molecules, and depend on the migration state and the cell differentiation may appear in different subcellular compartments (31).

In the epithelial cell cancers, reducing cell–cell adhesion leads to the cell migration and invasiveness and metastatic tumor spread (32). Transmembrane proteins regulate the para-cellular pathway and restrict the entrance of the pathogens. Thus, barrier function is one of the most prominent functions of the skin. Occludin is essential for the tight junction formation where occludin-deficient mice had defect in the skin barrier^16^. Therefore, dwon-regulation of the occludin decreases the barrier function of the tight junctions in the intestinal mucosa and breast (33). 

The loss of cell junctional sealing could involve in infiltration, proliferation, and transformation of the cancer cells and epithelial-mesenchymal transition in association with metastasis (34, 35). In certain tumors, loss of occludin was associated with carcinogenesis. A frequent complete loss of occludin was reported in cutaneous squamous cell carcinoma as compared to the precursor lesions and sun-exposed skin, which might result in decreased epithelial cell-cell adhesion and reduction of susceptibility to apoptosis (28).

Down-regulation of occludin expression was reported in ESCC as compared with the adjacent non-neoplastic specimens. Low expression of occludin was correlated with high histological grade and short overall survival. Also, the loss of occludin expression was associated with the poor prognosis in ESCC, and occludin expression levels was potentially a good predictor of prognosis in ESCC (21).

Breast cancer with the bone metastasis showed significantly lower occludin expression in comparison with those without bone metastasis (36). 

Loss of occludin protein was also observed in the gallbladder adenocarcinoma (37), poorly differentiated carcinoma from stomach and colon (38, 39) and cholangiocarcinoma (40) as compared with the adjacent normal tissues and specific benign lesions.

Recombinant S100A9, a calcium-binding protein A9, which is known as migration inhibitory factor has been shown to reduce the A-375 melanoma cell line proliferation and down-regulate *OCLN *gene expression in a time- and dose-dependent manner (41). 

Occludin has a pivotal role in the stabilization of the cellular connection and inhibition of apoptosis in natural cells (42).Thus, occludin, as an important component of TJ proteins, may be used as a target for the therapeutic interventions.

We can conclude that low expression of occludin in the patients with melanoma is a hallmark of cancer progression and may be used as a prognostic factor. Further research is needed to understand how molecular abnormalities in the expression of tight junction proteins could contribute to the tumorigenesis.
